# Supplementary material for: Neural correlates of perceptual consciousness from within: A narrative review of human intracranial research
Source: eLife. 2026 Jul 30;15:RP109604. doi: 10.7554/eLife.109604 (PMC13435562; doi:10.7554/eLife.109604)
Supplement: Peer review history

# Editor report: eLife Assessment

**DOI:** 10.7554/eLife.109604.4.sa0

**Authors:** Clare Press

**Affiliations:** University College London United Kingdom

This Review Article provides a scholarly, clear and well-structured review of intracranial research into the neural correlates of consciousness (NCCs). To our knowledge this is the first such review and is therefore likely to become a must-read for anyone working in the field of consciousness research. The authors discuss the difficulties that researchers must face when studying NCCs and how insights may emerge via intracranial recordings in humans. This no doubt reflects an in-depth, timely, and insightful contribution to the literature.

# Referee report: Reviewer #1 (Public review)

**DOI:** 10.7554/eLife.109604.4.sa1

[Editors' note: this version has been assessed by the Reviewing Editor without further input from the original reviewers. The authors have addressed the comments raised in the previous round of review.]

Summary:

In this review paper, the authors describe the concept of neural correlates of consciousness (NCC) and explain how noninvasive neuroimaging methods fall short of being able to properly characterise an unconfounded NCC. They argue that intracranial research is a means to address this gap and provide a review of many intracranial neuroimaging studies that have sought to answer questions regarding the neural basis of perceptual consciousness.

Strengths:

The authors have provided an in-depth, timely, and scholarly contribution to the study of NCCs. First and foremost, the review surveys a vast array of literature. The authors synthesise findings such that a coherent narrative of what invasive electrophysiology studies have revealed about the neural basis of consciousness can be easily grasped by the reader. The authors also succeed in describing how single-cell recordings can interface with task-design to help mitigate the impact of confounded neural activity when searching for NCCs.

The review is also, to the best of my knowledge, the first review to specifically target intracranial approaches to consciousness and to describe their results in a single article. This is a credit to the authors - as it becomes ever harder to apply strict tests to theories of consciousness using methods such as fMRI and M/EEG, it is important to have informative resources describing the results of human intracranial research so that theorists will have to constrain their theories further in accordance with such data. Additionally, the authors provide a compelling case for single-celled research in consciousness science, despite the dominance of theories situated at the system and circuit level of analysis. As far as the authors were aiming to provide a complete and coherent overview of intracranial approaches to the study of NCCs, I believe they have achieved their aim.

Weaknesses:

Overall, I feel positive about this paper. The authors have addressed my comments from my previous review and I see no significant weaknesses in the current version.

Comment on previous version:

No comments - congratulations to the authors!

# Referee report: Reviewer #2 (Public review)

**DOI:** 10.7554/eLife.109604.4.sa2

Summary:

In this work, the authors review the study of the neural correlates of consciousness (NCCs). They discuss several of the difficulties that researchers must face when studying NCCs, and argue that several of these difficulties can be alleviated by using intracranial recordings in humans.

They describe what constitutes an NCC, and the difficulties to distinguish between an NCC proper from the prerequisites and consequences of conscious processing.

They also describe the two main types of experimental designs used to study NCCs. These are the contrastive approach (with its report and non-report variants), and the supraliminal approach, each with their own merits and pitfalls.

They discuss the limitations of non-invasive methods, such as fMRI, EEG and MEG, as well as the limitations of the use of invasive recordings in non-human animals.

After setting the stage in this way, the authors provide an extensive review on the knowledge acquired by using invasive recordings in humans. This included population level measurements in vision and in other sensory modalities, as well as single neuron level studies. The authors also discuss studies of subcortical NCCs.

The second half of this work discusses the theoretical insights gained through the use of intracranial recordings, as well as their limitations, and a perspective for future work.

Strengths:

This work offers an impressive review, which will serve as a useful reference document, both for newcomers to the study of NCC as for experienced researchers. The inclusion of non-visual and subcortical NCCs is of particular merit, as these have been understudied.

Besides serving as a review, this work includes a perspective, exploring several directions to pursue for the progress of the field.

Weaknesses:

No major weaknesses.

Appraisal of whether the authors achieved their aims:

In this work, the authors have gathered an impressive review, and have discussed several important problems in the field of study of NCCs, as well as provided a perspective on how the field could move forward.

Discussion of the likely impact of the work on the field:

This work has the potential of becoming a must read for anyone working in the field of consciousness research.

Comment on previous version:

The authors have addressed all my concerns. Once again, my compliments for a nice piece of work.

# Referee report: Reviewer #3 (Public review)

**DOI:** 10.7554/eLife.109604.4.sa3

Summary:

This narrative review provides a clear, well-structured, and comprehensive synthesis of intracerebral recording work on the neural correlates of consciousness. It is written in an accessible manner that will be useful to a broad community of researchers, from those new to iEEG to specialists in the field.

Strengths:

The manuscript successfully integrates methodological and theoretical perspectives and offers a balanced overview of current sometimes contradicting evidence. As such, the manuscript is important as call for a concernted better exploration of NCCs using iEEG in the future.

Comments on latest version:

The current version of the manuscript is clear and complete. Kudos to the authors for their thorough revisions.

# Author comment: Author response

**DOI:** 10.7554/eLife.109604.4.sa4

**Authors:** François Stockart, Alexis Robin, Hal Blumenfeld, Milan Brázdil, Philippe Kahane, Liad Mudrik, Jasmine Thum, Michael Pereira, Nathan Faivre

**Affiliations:** Yale School of Medicine New Haven United States; Université Grenoble Alpes Grenoble France; Yale University New Haven United States; Masaryk University Brno Czech Republic; Centre Hospitalier Universitaire de Grenoble Grenoble France; Tel Aviv University Tel Aviv Israel; university of alabama Birmingham birmingham United States; Inserm La Tronche France; Centre National de la Recherche Scientifique Grenoble France

The following is the authors’ response to the previous reviews

**Reviewer 3 (Public review):**

Comments on revised version:

The current version of the manuscript is clear and complete. Kudos to the authors for their thorough revisions. My only remaining point concerns the definition of "report": "We define a report as any explicit behavioral response (whether verbal, manual, or otherwise) that communicates a participant's subjective state." It would be helpful to clarify whether this definition is intended to exclude purely internal, explicit self-reports that are not externally expressed. As currently formulated, the definition appears to require overt behavioral communication. However, this raises a conceptual issue in relation to the no-report paradigm literature, where the distinction between report, metacognitive access, and overt motor/verbal expression is precisely at stake.

Could the authors specify whether "report" is meant to (i) be restricted to externally observable, behaviorally expressed reports, or (ii) extend to internally generated, explicit metacognitive judgments even when they are not communicated? Clarifying this point would help situate the manuscript more precisely within ongoing debates on the role of report in identifying neural correlates of consciousness.

We thank the reviewer for prompting us to make this subtle but important distinction explicit. We agree that the two senses of "report", i.e., (i) externally observable, behaviorally expressed reports and (ii) internally generated, explicit metacognitive judgments that are not communicated, are conceptually distinct and that this distinction is precisely at stake in the no-report paradigm literature. We fully agree that sense (ii) (disentangling NCCs from covert metacognitive access) would be a valuable direction for future research. However, because the intracranial studies reviewed in the manuscript focus exclusively on distinguishing NCCs from overt behavioral reports, our definition is intentionally restricted to sense (i).

To clarify this point in the manuscript, we added the following sentence at lines 111–114:

"Note that the no-report intracranial studies described here attempt to distinguish NCCs from externally observable, behaviorally expressed reports, and not from internally generated metacognitive judgments that are not communicated."
